# Mycosis fungoides and Sézary syndrome: a population-wide study on prevalence and health care use in Finland in 1998–2016

**DOI:** 10.1186/s12913-021-06109-9

**Published:** 2021-02-22

**Authors:** Jaana Keto, Sonja Hahtola, Miika Linna, Liisa Väkevä

**Affiliations:** 1 Faculty of Medicine, University of Helsinki, Helsinki, Finland; 2 Department of Dermatology and Allergology, University of Helsinki and Helsinki University Central Hospital, Helsinki, Finland; 3 Institute of Healthcare Engineering, Management and Architecture, Aalto University, Espoo, Finland

**Keywords:** Cutaneous T-cell lymphoma, Health care use, Mycosis fungoides, Sézary syndrome

## Abstract

**Background:**

Information about health care use and costs of cutaneous T-cell lymphoma (CTCL) patients is limited, particularly in a European setting.

**Methods:**

In this population-wide study we set out to investigate prevalence, and trends in health care use in two CTCL subtypes, mycosis fungoides (MF) and Sézary syndrome (SS) over a time period of 19 years in 1998–2016 by using a nation-wide patient register containing data on all diagnosed MF and SS cases in Finland.

**Results:**

The prevalence of diagnosed MF and SS rose from 2.04 to 5.38/100000, and from 0.16 to 0.36/100000 for MF and SS respectively during 1998–2016. We found a substantial decrease in inpatient treatment of MF/SS in the past two decades with a mean of 2 inpatient days/patient/year due to MF/SS in 2016, while the mean numbers of MF/SS related outpatient visits remained stable at 8 visits/year/patient. Most MF/SS-related outpatient visits occurred in the medical specialty of dermatology. In a ten-year follow-up after MF/SS diagnosis, the main causes for outpatient visits and inpatient stays were MF/SS itself, other cancers, and other skin conditions. Also cardiovascular disease and infections contributed to the number of inpatient days. Mean total hospital costs decreased from 11,600 eur/patient/year to 3600 eur/patient/year by year 4 of the follow-up, and remained at that level for the remainder of the 10-year follow-up. MF/SS accounted for approximately half of the hospital costs of these patients throughout the follow-up.

**Conclusions:**

The nearly 3-fold increase in prevalence of diagnosed MF/SS during 1998–2016 puts pressure on the health care system, as this is a high-cost patient group with a heavy burden of comorbidities. The challenge can be in part answered by shifting the treatment of MF/SS to a more outpatient-based practice, and by adapting new pharmacotherapy, as has been done in Finland.

**Supplementary Information:**

The online version contains supplementary material available at 10.1186/s12913-021-06109-9.

## Background

Cutaneous T cell lymphomas (CTCL) are a heterogenous group of non-Hodgkin lymphomas characterized by skin homing T cells. The most common type of CTCL is mycosis fungoides (MF) accounting for around 60% of new cases [1]. Sézary syndrome (SS) accounts for 5% of CTCL cases. These two CTCL types have often been grouped together in literature [1, 2]. MF/SS has an annual incidence of 5.6 per 100,000 person-years and the disease more commonly affects males [3]. The evolution and prognosis of the disease is dependent on the stage of the disease. The five-year over-all survival rate of CTCL patients is over 80% in the least advanced disease stages 1A and 1B, but worsens with tumour progression, and nodal and blood involvement, declining to 18% for the most severe clinical stages IVA2 and IVB [2].

There are many therapeutic options available for the treatment of CTCL, with which long-term remissions can be achieved [1]. The less advanced stages of MF are usually treated with skin directed therapies, e.g. topical corticosteroids and UV therapy to relieve symptom burden. These treatment modalities require frequent outpatient visits in hospitals. The advanced stages of MF are treated with systemic therapies alone or in combination with skin directed therapy, or radiotherapy. Systemic therapies for MF include chemotherapy, monoclonal antibodies, extra-corporeal photoimmunotherapy, or biological response modifiers such as interferon, retinoids, rexinoids and denileukin diftitox [1]. Many forms of systemic therapy require hospitalisation of the patient, followed by frequent outpatient visits.

CTCL patients have many comorbidities, both physical and psychiatric. CTCL patients are in increased risk of developing diabetes mellitus type 2, cardiovascular disease, a secondary cancer, or depression [4, 5]. There is limited information about the role of these comorbidities in the total health care use of CTCL patients [6, 7]. Furthermore, the health care costs of CTCL and its comorbidities have not been reported for nationwide samples using standard health economic evaluation methods in a European setting.

As treatment options for MF/SS is an active field of study, information on the total health care use and its costs in this patient group may offer some beneficial context. The purpose of this study was to examine health care use among MF and SS patients in Finland: patterns in inpatient stays and outpatient visits, as well as the medical specialty in which MF/SS has been treated. In order to capture also the role of comorbidities in the total health care use of MF/SS patients, we formed a cohort of incident MF/SS patients and inspected their total health care use during 10 years following diagnosis.

## Methods

This study is a retrospective, population-wide non-intervention study on mycosis fungoides and Sézary syndrome (ICD-10: C84.0 and C84.1) in Finland. The data were collected from the national discharge register held by the National Institute for Health and Welfare. The discharge register covers all outpatient and inpatient secondary health care utilisation for the entire population of Finland (~ 5.6 million) and includes unique personal identification numbers for individuals which can be used to calculate the exact number of individual patients. The discharge register is regularly audited for completeness and accuracy [8]. The stage of MF/SS cannot be identified from the national discharge register.

The methods used in the present study include i) calculating the annual cross-sectional prevalence of diagnosed MF and SS in 1998–2016, and the respective use of health care services to treat MF/SS (outpatient visits, hospital admissions, bed-days), and ii) incident pooled cohorts in 2000–2005 and the use and corresponding costs of health care services for any cause in a follow-up period of 10 years.

Prevalence was calculated as point prevalence for each calendar year separately, using the size of the Finnish population on Dec 31st of each year as the denominator. Population size was acquired from the population register maintained by Statistics Finland. Deaths of MF/SS patients during the calendar year were not captured. Diagnosed prevalence was defined as the number of unique patients who, during the calendar year, had outpatient or inpatient health care contacts where the primary diagnosis or secondary diagnoses included one of the following ICD-10 codes:

C84.0 (mycosis fungoides), or

C84.1 (Sézary syndrome).

In order for the patient to be classified as incident, they could not have previous C84.0 or C84.1 related health care use in 5 years prior to their index date. Index date was defined as the first date when the patient had an outpatient or inpatient health care contact where C84.0 or 84.1 was the primary or secondary diagnosis. However, the incident patients were allowed to have prior health care contacts for other subtypes of C84. This rule permits the inclusion of patients who progressed for instance from mycosis fungoides to Sézary syndrome. MF and SS were grouped together due to the low number of patients with SS, as has been done also in previous CTCL literature [1, 2].

Using register linkage, all health care use for the prevalent and incident patient cohorts were collected. The health care services included outpatient and inpatient care in hospitals and inpatient community (primary) care. In our analyses, we grouped all health care utilisation and costs into several separate categories in the 10-year follow-up: 1) the total health care utilisation and costs, 2) MF/SS related health care utilisation and costs 3) total health care utilisation and costs according to comorbidity such as other cancer (C*), pneumonia and other infections (J18* and A*), Disease of the skin and subcutaneous tissue (L00*-L99*), Arthrosis (M15*-M19*), Cardiovascular disease (I2*-I6*), Depression (F32*-F33*), and other diagnoses. In addition, all health care use and costs were grouped according to medical specialty: oncology, dermatology, internal medicine, other specialty.

The costs for hospitalisations and hospital outpatient visits due to any cause were based on the NordDRG patient grouping definitions which use the International Classification of Diseases 10th revision (ICD-10) codes and the Finnish version of the Nordic Classification of Surgical Procedures (NCSP) codes for diagnostic and treatment procedures [9, 10]. The DRG cost weights for hospitalisations and outpatient visits were based on individual-level cost accounting data from several hospitals, of which a national average was calculated and used in this study. The unit cost estimates for community care bed-days were derived from the national price list for unit costs of health care services in Finland [11]. The cost of medication administered in the hospital is included in the in the unit costs. For all cost analyses, we used the 2014 national average price level and the 2014 national standard price list, which was the latest version available.

## Results

**Fig. 1:**
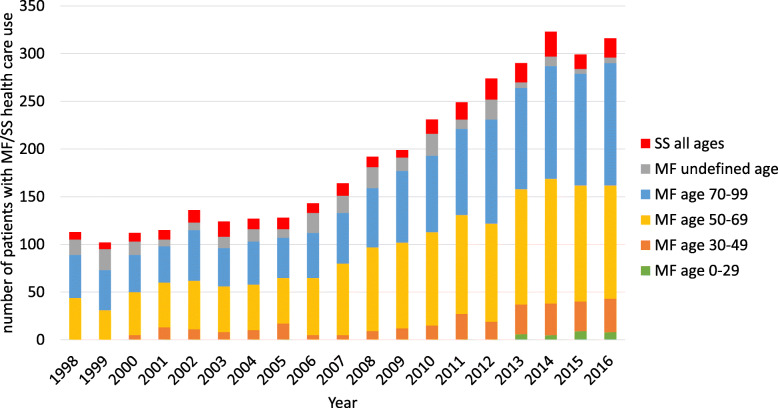
Age distribution among all diagnosed Finnish MF/SS patients in 1998–2016

Between 1998 and 2016, the prevalence of diagnosed mycosis fungoides rose from 2.04/100000 to 5.38/100000, and the prevalence of diagnosed Sézary syndrome rose from 0.16/100000 to 0.36/100000 (Table S1, Additional file 1). Age distribution among MF/SS patients in 1998–2016 is presented in Fig. 1. Prevalence estimations for MF by sex and more detailed age group can be found in Table S2, Additional file 1.

### Hospital treatment of mycosis fungoides and Sézary syndrome

**Fig. 2:**
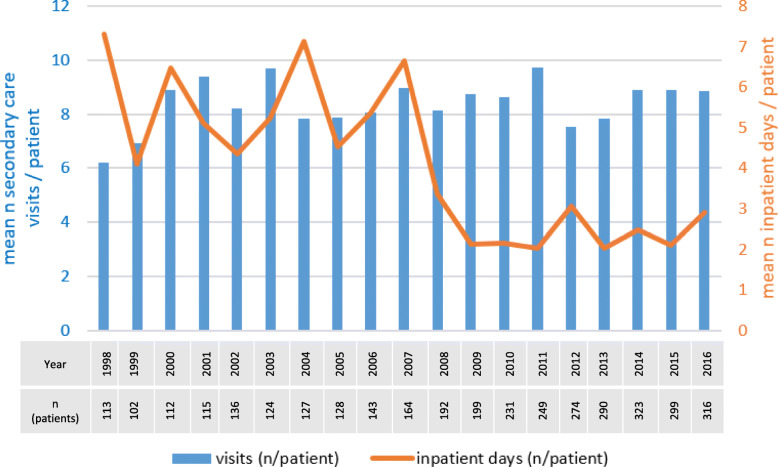
Hospital treatment of mycosis fungoides and Sézary syndrome patients. The blue bars represent the mean number of hospital outpatient visits due to MF/SS per annum per patient, and the orange line represents the mean number of inpatient days due to MF/SS per annum per patient

Inpatient treatment of MF/SS has decreased in the past two decades (Fig. 2). A notable decrease in inpatient days per patient can be observed between the year 2007 and 2009, when the inpatient days drop by two thirds from 6.7 to 2.1. Meanwhile, the mean number of hospital outpatient visits for MF/SS per patient has remained constant - MF/SS patients have, on average, 8 annual hospital visits related to their disease.

**Fig. 3:**
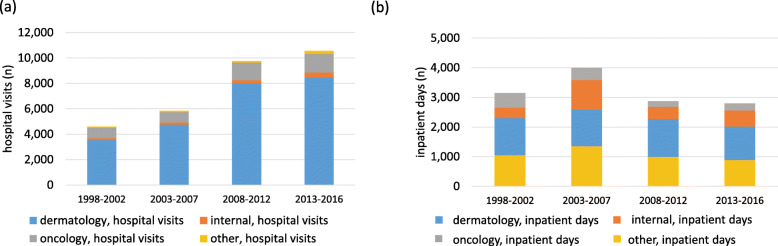
MF/SS treatment by medical specialty. Hospital visits where the primary diagnosis was MF/SS are presented in figure **a**), inpatient days in figure **b**)

MF/SS related health care use by specialty in which the patient was treated are presented in Fig. 3. Most MF/SS-related hospital visits occurred in dermatology, where their number grew notably during the 18-year inspection period (Fig. 3a). Most MF/SS-related inpatient days took place either in dermatology or in “other” specialty (Fig. 3b). Some 57% of inpatient days in the “other” specialty group occurred in a hospital, the rest in a primary care inpatient ward.

### Total health care use in a 10-year follow-up after MF/SS diagnosis

A cohort was formed of all patients diagnosed with MF/SS in 2000–2005 (*n* = 247). Ten years after diagnosis, 101 (41%) of these patients were still using hospital services for any reason.

**Fig. 4:**
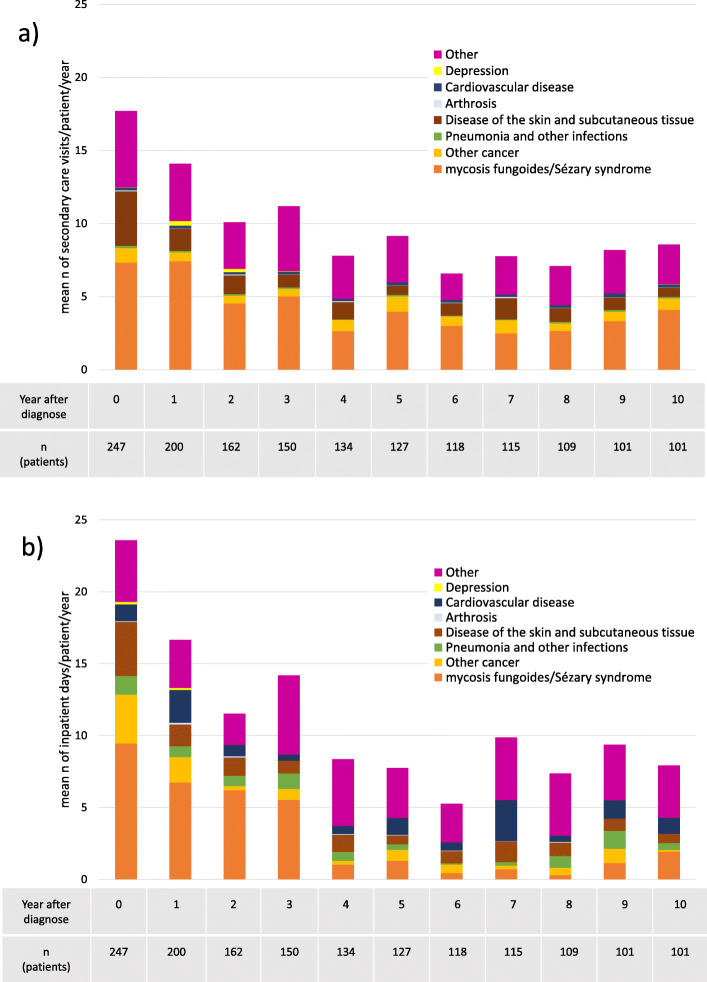
Total secondary health care use by cause among MF/SS patients in a 10-year follow-up after MF/SS diagnosis. Mean n of annual hospital visits are presented in figure **a**), inpatient days in figure **b**)

During the 10-year follow-up, the average number of annual hospital outpatient visits per patient decreased by 50% from 18 to 9 (Fig. 4a). MF/SS was the cause for 40% of all visits in the inspected cohort during year zero, i.e. year of MF/SS diagnosis. The proportion remained relatively similar throughout the follow-up period. Besides MF/SS, other common reasons for hospital visits were other cancer and disease of the skin and subcutaneous tissue.

The average number of annual inpatient days per patient decreased from 24 to 8 during the 10-year follow-up (Fig. 4b). MF/SS was the main cause for inpatient days during the first 4 years of the follow-up period, after which MF/SS related inpatient days decreased drastically in both absolute and relative terms. Other common reasons for hospitalisations were disease of the skin and subcutaneous tissue, other cancer, cardiovascular disease, and pneumonia and other infections.

### Health care costs of MF/SS patients in a 10-year follow-up

The health care costs (hospital outpatient visits and inpatient days) of MF/SS patients are presented as mean costs in euros (€) per patient per year in Table 1. The total costs were highest at the year of MF/SS diagnosis, after which they declined until reaching a steady state at year four. Costs for MF/SS related health care use represented about half of the total health care use of these patients throughout follow-up.

**Table 1:** Mean hospital treatment costs of MF/SS patients in 10-year follow-up after diagnosis

Year	n (MF)	n (SS)	MF/SS treatment costs (mean €/patient)	Other disease costs (mean €/patient)	Mean MF/SS share of total costs (%)
0	207	40	11,630	11,107	51
1	173	27	9804	6619	60
2	147	15	5854	4136	59
3	135	15	6194	5284	54
4	119	15	2700	3751	42
5	112	15	3928	4078	49
6	103	15	2892	3298	47
7	100	15	2485	5288	32
8	94	15	2632	4283	38
9	86	15	3613	4720	43

## Discussion

This nation-wide study describes trends in health care utilisation among MF/SS patients during a 20-year period in Finland. We are not aware of previous studies with similar coverage, both population- and time-wise. We report a nearly three-fold rise in prevalence of MF/SS in 1998–2016, which could be due to the same reasons as the increasing incidence trends in cutaneous T-cell lymphoma observed in Norway, and possibly in the United States of America as well, although the U.S. health care system and data sources available are different from the ones used in the present study [3, 12]. During the past two decades, the biggest change in the treatment of MF/SS patients has been in the mean number of MF/SS-related inpatient days, which decreased to one third between 2007 (6.7 annual inpatient days per patient) and 2009 (2.1 annual inpatient days per patient). There has been a general tendency to treat patients with chronic diseases as outpatients instead of hospitalisation in Finland, which could partly explain this finding. MF/SS specific treatment practices in Finland have also changed in the past two decades: previously, patients undergoing phototherapy or skin-directed therapy were usually hospitalised in the beginning of the new treatment. More recently these treatments have been performed in an outpatient manner. Also, the use of bexarotene, which does not require inpatient monitoring of the patient, has become widespread as a first-line systemic therapy for MF/SS [13]. Further research would be needed to support these potential explanations for the decrease in inpatient care of MF/SS patients.

Unlike inpatient stays, the number of outpatient visits has remained constant for the past two decades, on average eight annual visits per patient. In clinical practice these visits typically include the regular follow-up visits (three or four per year depending on the stage of the disease) as well as the visits due to the sudden exacerbations of the lymphoma and other acute illnesses. We assume that inpatient treatment has been to some extent replaced by outpatient visits. Interestingly, this has not lead to an increase in the mean number of outpatient visits. This may be due to improved outpatient-based treatment protocols, or improved disease control.

Most MF/SS-related hospital visits and inpatient days have occurred in dermatological clinics throughout the 20-year inspection period. A minority of hospital visits occurred in oncology. This usually happens in the later stages of the disease, when oncological treatment, e.g. chemotherapy is started.

MF/SS patients have been reported to have a high burden of comorbidities, in particular other cancers and depression [4, 5]. This was considered also in the design of the present study, as all secondary health care use of MF/SS patients was reported by cause for a cohort of 247 incident MF/SS patients for 10 years following diagnosis. The most common reasons for hospital visits and inpatient days among MF/SS patients were in line with existing comorbidity literature [4, 5]. Other cancers, disease of the skin and subcutaneous tissue, cardiovascular disease, and pneumonia and other infections were reflected especially in the number of inpatient days among MF/SS patients. The mean number of hospital visits during year of diagnosis for MF/SS (7.3) or for any reason including MF/SS (17.6) were in the same range with physician office visits recently reported in a claims database analysis from the United States, in which health care use during index year was observed for 1981 MF patients with the same health insurance company [6].

Health care costs were highest at the year of MF/SS diagnosis, after which they declined until reaching a steady stage at year four. The diagnosis of MF is challenging and the delay from the first symptoms to diagnosis is 4–6 years [14]. During the first year, the diagnostic procedures, including e.g. immunohistology, molecular cytogenetics and disease staging, make up a big part of the costs. The patient also needs more guidance and support in terms of frequent visits.

Previous information about the costs of treating MF/SS is limited, particularly in a European setting [6, 7]. As this study relied on standard methods of health economic evaluation - nationwide registers combined with national standard unit costs and patient grouping systems - health care use of MF/SS patients may now be compared with other patient groups to better understand the burden of disease. For instance, during the first 4 years after MF/SS diagnosis, the mean costs per patient are 8- to 16-fold compared with the general population of Finland [15]. After this, the mean numbers of hospital visits and inpatient days stabilise, which could be partially due to changes in the cohort population. It is reasonable to assume that the patients who were high utilisers of health care close to MF/SS diagnosis died sooner, while patients with a more indolent disease and less comorbidities remained in the cohort. Also, if the patient moves to a heavily supported environment such as a nursing home, their health care costs are transferred from the health care sector to the social sector and are thus not visible in the present study. In another Finnish dataset, the cumulative hazard for death or institutionalisation was 14% 1 year from MF/SS diagnosis, 19% 2 years from diagnosis, and 26% 3 years from diagnosis (*n* = 102) (Additional file 2). This is approximately similar to the loss to follow-up in the present study. A future prospect would be to further characterise health care use and cost distribution among MF/SS patients by identifying medication and treatment strategies which are associated with higher costs and morbidity.

The major uncertainty of the study relates to the quality of the data in the national discharge register held by the National Institute for Health and Welfare. While the discharge register is regularly checked for completeness and accuracy, there has been no MF/SS specific audit [8]. Despite its name, the discharge register includes information also on all patients who have been treated in an outpatient setting in the secondary health care sector, which is responsible for diagnosing and treatment of all cancers in Finland. It is thus reasonable to assume that all patients who have received a MF/SS diagnosis in Finland are represented in our data. However, as previous research reports a mean time of 4–6 years from the onset of symptoms to diagnosis of MF, the true prevalence could be somewhat higher than the diagnosed prevalence we report [14]. Another limitation of the study is the level of detail we are able to provide in terms of patient characteristics. As we only had access to aggregate level, grouped data on health care use among the total MF/SS patient population at any given year, we were unable to standardise our health care use results according to patient characteristics such as age and sex.

## Conclusions

In this paper, we have presented trends in prevalence, and secondary health care use of mycosis fungoides and Sézary syndrome patients in Finland over a time period of two decades. The nearly three-fold increase in prevalence of diagnosed MF/SS during 1998–2016 puts pressure on the health care system, as this is a high-cost patient group with a heavy burden of comorbidities. The challenge can be in part answered by shifting the treatment of MF/SS to a more outpatient-based practice, and by adapting new pharmacotherapy, as has been done in Finland.

## Supplementary Information

**Additional file 1 **Detailed prevalence of diagnosed MF/SS. **Table S1.** Number of diagnosed mycosis fungoides (MF) and Sézary syndrome (SS) patients using health care services during each calendar year, and the corresponding prevalence (1/100000) in Finland in 1998–2016. **Table S2a.** Number of diagnosed mycosis fungoides (MF) patients using health care services during each calendar year, and the corresponding prevalence (1/100000) among men by age group in Finland in 1998–2016. **Table S2b**. Number of diagnosed mycosis fungoides (MF) patients using health care services during each calendar year, and the corresponding prevalence (1/100000) among men by age group in Finland in 1998–2016.

**Additional file 2.** Cumulative hazard of death and institutionalisation after Mycosis fungoides or Sézary syndrome diagnosis.

## Data Availability

The data used in this study is available by request from The Finnish Institute of Health and Welfare.

## Abbreviations

CTCL: Cutaneous T-cell lymphoma
MF: Mycosis fungoides
SS: Sézary syndrome
