# Supplementary material for: Mycosis fungoides and Sézary syndrome: a population-wide study on prevalence and health care use in Finland in 1998–2016
Source: BMC Health Serv Res. 2021 Feb 22;21:166. doi: 10.1186/s12913-021-06109-9 (PMC7898452; doi:10.1186/s12913-021-06109-9)
Supplement: Supplementary file 1 — Additional file 1 Detailed prevalence of diagnosed MF/SS. Table S1. Number of diagnosed mycosis fungoides (MF) and Sézary syndrome (SS) patients using health care services during each calendar year, and the corresponding prevalence (1/100000) in Finland in 1998–2016. Table S2a. Number of diagnosed mycosis fungoides (MF) patients using health care services during each calendar year, and the corresponding prevalence (1/100000) among men by age group in Finland in 1998–2016. Table S2b. Number of diagnosed mycosis fungoides (MF) patients using health care services during each calendar year, and the corresponding prevalence (1/100000) among men by age group in Finland in 1998–2016. [file 12913_2021_6109_MOESM1_ESM.pdf]

Additional file 1. Detailed prevalence of diagnosed MF/SS.

Supplementary Table 1. Number of diagnosed mycosis fungoides (MF) and Sézary syndrome (SS) patients using health care services during each calendar year, and the corresponding prevalence (1/100 000) in Finland in 1998-2016.

	**Year**																		
	1998	1999	2000	2001	2002	2003	2004	2005	2006	2007	2008	2009	2010	2011	2012	2013	2014	2015	2016
Population Dec 31st	5159646	5171302	5181115	5194901	5206295	5219732	5236611	5255580	5276955	5300484	5326314	5351427	5375176	5401267	5426674	5451270	5471753	5487308	5503297
n SS patients	8	7	9	10	13	16	11	12	10	13	11	8	15	18	22	20	26	15	20
n MF patients	105	95	103	105	123	108	116	116	133	151	181	191	216	231	252	270	297	284	296
SS 1/100 000	0.16	0.14	0.17	0.19	0.25	0.31	0.21	0.23	0.19	0.25	0.21	0.15	0.28	0.33	0.41	0.37	0.48	0.27	0.36
MF 1/100 000	2.04	1.84	1.99	2.02	2.36	2.07	2.22	2.21	2.52	2.85	3.40	3.57	4.02	4.28	4.64	4.95	5.43	5.18	5.38

Supplementary Table 2a). Number of diagnosed mycosis fungoides (MF) patients using health care services during each calendar year, and the corresponding prevalence (1/100 000) among men by age group in Finland in 1998-2016.

	Year																		
	1998	1999	2000	2001	2002	2003	2004	2005	2006	2007	2008	2009	2010	2011	2012	2013	2014	2015	2016
**Age 40-49**																			
Popul.	407592	400855	395721	392833	389193	387763	385853	383001	380065	378447	377671	373253	368162	362194	355356	347351	342288	340102	338810
n MF	3	3	5	5	6	3	3	6	5	5	9	3	6	9	6	10	12	10	10
1/100000	0.61	0.62	1.26	1.27	1.54	0.64	0.65	1.57	1.32	1.32	2.38	0.67	1.63	2.48	1.69	2.88	3.51	2.94	2.95
**Age 50-59**																			
Popul.	340707	354432	370007	374532	390373	398561	405168	404641	400700	394668	386983	381713	377765	375750	373341	372917	372309	370591	368581
n MF	12	12	16	17	17	20	16	15	18	12	20	24	23	24	21	30	27	23	22
1/100000	3.52	3.39	4.32	4.54	4.35	5.02	3.95	3.71	4.49	3.04	5.17	6.29	6.09	6.39	5.62	8.04	7.25	6.21	5.97
**Age 60-69**																			
Popul.	224567	227778	226895	235819	235026	240655	246587	258721	274834	290648	304745	317435	331591	335965	350189	357877	364408	364669	361856
n MF	14	8	15	18	21	16	18	20	23	32	33	35	34	38	36	43	55	60	59
1/100000	6.23	3.51	6.61	7.63	8.94	6.65	7.30	7.73	8.37	11.01	10.83	11.03	10.25	11.31	10.28	12.02	15.09	16.45	16.30
**Age 70-79**																			
Popul.	136730	142741	147108	151283	154538	156356	158858	161895	164251	167432	171389	175443	175962	184527	184707	189963	195270	206094	220036
n MF	15	14	13	15	18	10	16	17	25	26	28	36	36	41	51	44	47	46	51
1/100000	10.97	9.81	8.84	9.92	11.65	6.40	10.07	10.50	15.22	15.53	16.34	20.52	20.46	22.22	27.61	23.16	24.07	22.32	23.18
**Age 80-89**																			
Popul.	42287	41818	43554	45196	46818	49359	52384	55900	59313	62694	66831	70587	73871	77226	79683	81672	83834	86563	88708
n MF	11	10	9	6	8	10	6	6	3	6	8	14	18	22	24	23	29	28	26
1/100000	26.01	23.91	20.66	13.28	17.09	20.26	11.45	10.73	4.21	9.57	11.97	19.83	24.37	28.49	30.12	28.16	34.59	32.35	29.31

Supplementary Table 2b). Number of diagnosed mycosis fungoides (MF) patients using health care services during each calendar year, and the corresponding prevalence (1/100 000) among women by age group in Finland in 1998-2016.

	Year																		
	1998	1999	2000	2001	2002	2003	2004	2005	2006	2007	2008	2009	2010	2011	2012	2013	2014	2015	2016
**Age 40-49**																			
Popul.	395441	390076	385971	383815	380451	379001	376337	373876	371257	369516	368433	363842	358598	352557	345412	336755	331708	328146	325754
n MF	3	3	3	8	5	8	10	6	3	3	3	7	9	7	7	9	9	9	10
1/100000	0.63	0.64	0.65	2.08	1.31	2.11	2.66	1.60	0.67	0.68	0.68	1.92	2.51	1.99	2.03	2.67	2.71	2.74	3.07
**Age 50-59**																			
Popul.	341511	353328	368223	372072	387710	396594	404652	405220	401659	396261	389204	384489	381200	379672	377025	376254	374173	372020	369578
n MF	9	6	7	5	7	7	8	5	12	17	15	13	17	17	18	21	20	15	15
1/100000	2.64	1.70	1.90	1.34	1.81	1.77	1.98	1.23	2.99	4.29	3.85	3.38	4.46	4.48	4.77	5.58	5.35	4.03	4.06
**Age 60-69**																			
Popul.	257149	258603	255545	263477	261169	265820	270711	281690	297295	312447	326283	337770	352118	355914	370927	379527	387436	388254	385074
n MF	9	5	7	7	6	5	6	8	7	14	20	18	24	25	28	27	29	24	23
1/100000	3.50	1.93	2.74	2.66	2.30	1.88	2.22	2.84	2.35	4.48	6.13	5.33	6.82	7.02	7.55	7.11	7.49	6.18	5.97
**Age 70-79**																			
Popul.	219904	226209	227440	227546	227415	224838	224575	224389	224364	225574	227054	229271	227170	235234	233171	237513	242027	252265	266621
n MF	13	13	11	9	18	15	18	13	13	13	19	15	16	18	20	16	19	12	22
1/100000	5.91	5.75	4.84	3.96	7.92	6.67	8.02	5.79	5.79	5.76	8.37	6.54	7.04	7.65	8.58	6.74	7.85	4.76	8.25
**Age 80-89**																			
Popul.	109269	107576	110935	114133	116616	120283	123903	128450	132614	136022	139967	145070	147738	149392	150579	150173	150657	151376	151974
n MF	6	5	6	8	9	5	5	6	9	8	7	10	10	9	14	18	18	19	18
1/100000	5.49	4.65	5.41	7.01	7.72	4.16	4.04	4.67	6.79	5.88	5.00	6.89	6.77	6.02	9.30	11.99	11.95	12.55	11.84
