# Supplementary material for: Mycosis fungoides and Sézary syndrome: a population-wide study on prevalence and health care use in Finland in 1998–2016
Source: BMC Health Serv Res. 2021 Feb 22;21:166. doi: 10.1186/s12913-021-06109-9 (PMC7898452; doi:10.1186/s12913-021-06109-9)
Supplement: Supplementary file 2 — Additional file 2. Cumulative hazard of death and institutionalisation after Mycosis fungoides or Sézary syndrome diagnosis. [file 12913_2021_6109_MOESM2_ESM.pdf]

Additional file 2. Cumulative hazard of death and institutionalisation after Mycosis fungoides or Sézary syndrome diagnosis.

Data: The Opioid Patient Trends in Finland OPTI dataset includes full data on social and health care use, prescribed medications, and deaths for all inhabitants in Finland with reimbursed opioid medication in 2009-2017 (n = 1 761 584).

Methods: The OPTI dataset was investigated for patients who had secondary health care outpatient visits or inpatient stays where the primary or secondary diagnosis was ICD-10 C84.0 (mycosis fungoides MF) or C84.1 (Sézary syndrome SS) for the first time in 2012-2014. The patients had a three-year wash-out period, during which they could not have MF/SS related health care use. These incident patients were followed up for three years after the initial MF/SS diagnosis to inspect the cumulative hazard of death and institutionalisation, which was defined as moving to a nursing home or a similar social care institution.

Results: 102 individuals with a MF or SS diagnosis were identified. The cumulative hazard for death or institutionalisation is presented in table 1.

Supplementary Table 3. Cumulative hazard of death and institutionalisation in a sample of 102 incident MF/SS patients.

	Year 1	Year 2	Year 3
Death % (n)	10.8% (11)	14.7% (15)	21.6% (22)
Institutionalisation % (n)	2.9% (3)	3.9% (4)	4.9% (5)
Death or institutionalisation % (n)	13.7% (14)	18.6% (19)	26.5% (27)
